# Zinc Oxide/Phosphorus-Doped Carbon Nitride Composite as Potential Scaffold for Electrochemical Detection of Nitrofurantoin

**DOI:** 10.3390/bios12100856

**Published:** 2022-10-10

**Authors:** Faheem Ahmed, Thangavelu Kokulnathan, Ahmad Umar, Sheikh Akbar, Shalendra Kumar, Nagih M. Shaalan, Nishat Arshi, Mohd Gulfam Alam, Abdullah Aljaafari, Adil Alshoaibi

**Affiliations:** 1 Department of Physics, College of Science, King Faisal University, P.O. Box 400, Al-Ahsa 31982, Saudi Arabia; 2 Department of Electro-Optical Engineering, National Taipei University of Technology, Taipei 106, Taiwan; 3 Department of Chemistry, Faculty of Science and Arts and Promising Centre for Sensors and Electronic Devices (PCSED), Najran University, Najran 11001, Saudi Arabia; 4 Department of Materials Science and Engineering, The Ohio State University, Columbus, OH 43210, USA; 5 Department of Physics, School of Engineering, University of Petroleum & Energy Studies, Dehradun 248007, India; 6 Physics Department, Faculty of Science, Assiut University, Assiut 71516, Egypt; 7 Department of Basic Sciences, Preparatory Year Deanship, King Faisal University, P.O. Box 400, Al-Ahsa 31982, Saudi Arabia; 8 Department of Chemistry, Faculty of Science, Islamic University of Madinah, Madinah 42351, Saudi Arabia

**Keywords:** metal oxide, carbonous material, heteroatom, antibiotic medication

## Abstract

Herein, we present an electrocatalyst constructed by zinc oxide hexagonal prisms/phosphorus-doped carbon nitride wrinkles (ZnO HPs/P-CN) prepared via a facile sonochemical method towards the detection of nitrofurantoin (NF). The ZnO HPs/P-CN-sensing platform showed amplified response and low-peak potential compared with other electrodes. The exceptional electrochemical performance could be credited to ideal architecture, rapid electron/charge transfer, good conductivity, and abundant active sites in the ZnO HPs/P-CN composite. Resulting from these merits, the ZnO HPs/P-CN-modified electrode delivered rapid response (2 s), a low detection limit (2 nM), good linear range (0.01–111 µM), high sensitivity (4.62 µA µM^−1^ cm^2^), better selectivity, decent stability (±97.6%), and reproducibility towards electrochemical detection of NF. We further demonstrated the feasibility of the proposed ZnO HPs/P-CN sensor for detecting NF in samples of water and human urine. All the above features make our proposed ZnO HPs/P-CN sensor a most promising probe for detecting NF in natural samples.

## 1. Introduction

Water is an indispensable and precious reserve for every living entity to sustain life in this world [1,2,3]. With human activities and the industrial revolution, water sources have become contaminated with various types of pollutants, which bring serious harm to human beings and other organisms throughout the world [3]. The chief water contaminants are households, organic materials, drug manufacturers, large-scale animal feeding operations, organic materials, industries, and pharmaceutical residues [4,5,6]. Notably, the World Health Organization revealed that nearly 30% of individuals living rurally need hygienic water sources, and more than 2,000,000 humans die every year from contamination of drinkable water [7,8,9]. Among the different pollutants, the most notorious are pharmaceutical residues, which harm biodiversity. Every day, approximately 35 billion defined daily-dose pharmaceutical items are devoured around the world [9]. After their use, the improper disposal of unused, unwanted, and expired pharmaceutical compounds into the biosphere is the cause of pollution as well as other negative impacts [10,11]. Among the several types of pharmaceutical products, antibiotics have gained special attention due to their ability to develop antimicrobial resistance strains, their extensive usage, low degradation properties, and even causing death, if serious [12,13].

Antibiotics are medicines broadly used to treat or prevent bacterial infections in microorganisms as well as growth promoters in animal husbandry and aquaculture, which have been identified as significant pollutants in water [14,15]. Nitrofurantoin (NF) is one of the most commonly used nitrofuran antibiotics for the treatment of skin sensitizer, amebicide, fungicide, coccidiosis infections, bladder cancer, anti-inflammatory, and infections in the urinary tract [16,17,18,19,20,21,22,23,24]. Over use and the residues of NF produce a series of side effects such as pulmonary toxicity, neuropathy, flatulence, drowsiness, dizziness, numbness, hepatotoxicity, and other reactions. Even worse, the metabolic path of nitro-containing groups (NF) is reduced into the cytotoxic products (reactive oxygen species and NO• radicals) [25,26]. Hence, NF is often released into the environment foremost as an ecological system contagion, which can be hazardous even at a low-level amount [21,22,23,24,25]. On behalf of these reasons, many countries (United States, European Union, Australia, Brazil, China, Thailand, and the Philippines) have strictly prohibited the usage of NF due to its severe effects against all living organisms and the ecosystem. Roughly 1% of NF is eliminated as aminofurantoin in urine from original form [16].

Likewise, from the drug (expired pills) and trade production waste (residues) of NF that is directly discharged into local groundwaters, rivers, and soils, it may cause severe well-being effects in both human beings and other living microbes. Therefore, it is essential to stringently supervise the residual levels of NF in potable water and biological samples. The conventional methods such as colorimetry, photoluminescence, polarography, flow injection-spectrophotometry, surface-enhanced Raman spectroscopy, high-performance liquid chromatography, and flow injection-spectrophotometry were used for detecting NF [16,17,18,21,22,23,24,25,26,27,28,29,30]. Amongst the mentioned analytical methods, one of the most ideal platforms for the detection of trace amounts of molecules in food and environmental samples is the electrochemical sensor, due to their special features including high sensitivity, low cost, excellent selectivity, and portability [31,32,33,34]. However, the traditional electrode is usually limited by sluggish kinetics with low electrochemical performance and high overpotentials. Electrochemical sensor performances are dependent on electrocatalysts that play a vital role to produce amplified peak intensity and low-on-set peak potential.

In this sense, metal oxides (MOs) in the electrochemical sensors’ field have been considered promising electrocatalysts [35]. Amongst a wealth of MOs, zinc oxide (ZnO) has gained extensive attention as an electrode material for electrochemical sensors owing to its conspicuous advantages such as biocompatibility, tunable bandgap, non-toxicity, economic synthesis, and good catalytic activity [36,37,38,39]. Up to now, various types of morphology has been reported for ZnO such as spheres, pompons, mushrooms, rods, wires, cubes, aggregates, wrinkles, hexagonal prisms, plates, and so on [36,40,41,42,43]. In particular, the hexagonal prism’s structure may be a preferable candidate for electrochemical detection. Substantial research pursuits were devoted to improving the stability and conductivity, including ZnO incorporation into desirable conductive materials that could enhance the electrocatalytic activity. According to previous knowledge, the two-dimensional graphitic-carbon nitride (g-CN) with a unique property is regarded as a potential catalyst for electrochemical sensing [43,44,45].

Recently, non-metal heteroatom-doped g-CN has ignited tremendous research interests in electrochemical sensors owing to it modifying the physicochemical properties such as active sites, electronic conductivity, synergy, more defects, charge transfer mobility, high stability, its electronic structure, and excellent biocompatibility [46,47]. Phosphorus-doped g-CN could greatly modulate the physicochemical properties [48,49,50,51,52]. Based on the above statements, P-CN was chosen to be the supporting material for the electrochemical sensing. To date, no work was carried out on electrochemical sensing using P-CN-modified electrodes. Herein, a ZnO HPs/P-CN composite was designed and synthesized via a facile sonochemical method, and further utilized modified electrode material for NF sensing. The fabricated electrode presented excellent electrochemical activity to NF compared with the other electrodes. Moreover, the proposed electrochemical platform was productively applied to detect NF in biological and water samples with high accuracy.

## 2. Experimental Details

### 2.1. Chemicals and Reagents

Zinc acetate tetrahydrate (Zn(CH₃CO₂) ≥ 98%), hexamethylenetetramine (HMTA, (CH₂)₆N₄ ≥ 99%), ammonium fluoride (NH_4_F ≥ 98%), nitrofurantoin (NF), N, N-dimethylformamide, and melamine were purchased from Sigma Aldrich and Merck and used without further purification. All the electrochemical experiments were carried out using 0.05 M phosphate buffer (PB) as the supporting electrolyte.

### 2.2. Synthesis of P-CN

The wrinkled P-CN were prepared by thermal polymerization method [53]. Briefly, a definite quantity of C_3_H_6_N_6_ was dispersed into 50 mL DI water under vigorous stirring. With vigorous stirring, an appropriate amount of NH_4_H_2_PO_4_ was added to the aforesaid solution and kept for 1 h to prepare the homogenous mixture. The product was then collected by centrifugation and dried at 50 °C in the oven. Then, the product was transferred into the muffle furnace under an air atmosphere to heat for about 3 h at 550 °C. Finally, the P-CN was washed by DI water multiple times to get rid of unreacted impurities and was dried overnight. For comparison, g-CN was prepared without NH_4_H_2_PO_4_ in a similar process.

### 2.3. Synthesis of ZnO HPs

The ZnO HPs were prepared using the hydrothermal method [54,55]. In the classic synthesis, 0.1 M of Zn (CH₃CO₂)₂ was dissolved in 40 mL of DI water and stirred for 15 min. After that, 3 mmol of HMTA and 6 mmol of NH4F were slowly injected into the above solution under vigorous stirring and the mixture was stirred for 1 h. Next, the mixture was placed in a stainless-steel autoclave to carry out the hydrothermal reaction at 180 °C for about 12 h in an air oven. The precipitate was then collected by centrifugation, washed many times in ethanol and DI water, and dried overnight. Finally, the as-prepared ZnO HPs were calcined for a period of 3 h at 400 °C under air-atmosphere.

### 2.4. Synthesis of ZnO HPs/P-CN Composite

**Scheme 1:**
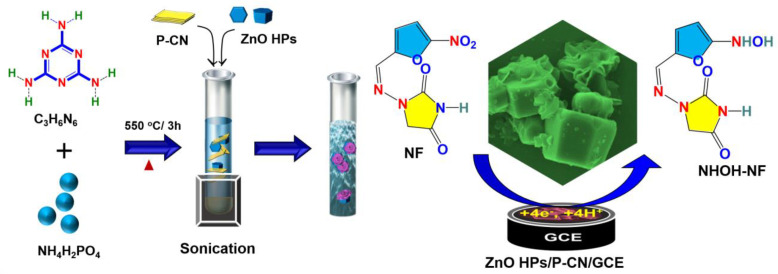
Schematic to prepare ZnO HPs/P-CN composite for electrochemical detection of NF.

The ZnO HPs/P-CN composite was synthesized via a facile sonochemical technique as schematically illustrated in Scheme 1. Briefly, ZnO HPs and P-CN were taken in 1:1 ratio and ultrasonicated for 3 h, followed by overnight drying at 50 °C to form a ZnO/P-CN composite.

### 2.5. Instrumentations and Methods

Phase identification was performed by using X-ray diffraction (XRD) using (XRD, Rigaku D/maxB, DMX-2200) and the Fourier transform infrared spectra in the range of 500–4000 cm^−1^ using a PerkinElmer IR spectrometer. The chemical state was examined via X-ray photoelectron spectroscopy (XPS, Thermo scientific multi-lab 2000, Waltham, MA, USA). The surface morphology was studied using scanning electron microscopy (JSM-6510series) and energy dispersive X-Ray (JEOL service advanced technology) spectroscopy. The electrochemical properties were explored using electrochemical impedance spectroscopy (EIS) through Autolab. CHI workstation was functional to carry out the electrochemical measurements in the three-electrode cell. The glassy carbon electrode (GCE) (surface area = 0.071 cm^2^), saturated Ag|AgCl, and Pt wire were active as working, reference, and counter electrodes, respectively.

### 2.6. Fabrication of Electrode

The bare GCE was polished with alumina slurries, continued by ultrasonic cleaning in DI water/ethanol mixture for a few minutes, and later rinsed with ample DI water. To prepare electrocatalyst ink, 4 mg of ZnO HPs/P-CN composite was dispersed in 1 mL DI water with the assistance of ultrasonication for about 30 min at room temperature. Then, 5 µL of ZnO HPs/P-CN ink was coated onto a GCE surface, and then dried at a certain temperature. A similar protocol was followed to fabricate ZnO HPs-, P-CN- and g-CN- modified GCEs for electrochemical comparison study.

## 3. Results and Discussion

### 3.1. Physical Characterization of ZnO HPs/P-CN Composite

**Figure 1:**
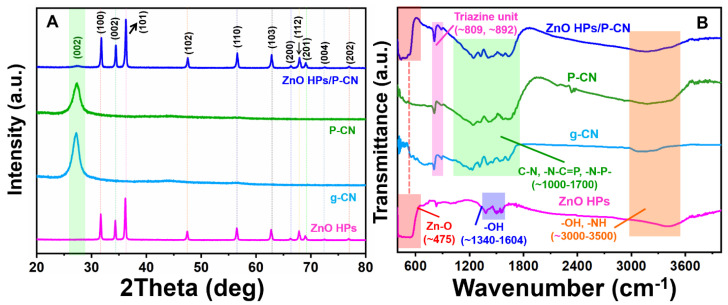
(**A**) XRD patterns and (**B**) FTIR spectra of ZnO HPs, g-CN, P-CN, and ZnO HPs/P-CN.

The crystallographic structure of the ZnO HPs/P-CN composite was obtained by an XRD technique (Figure 1A). The XRD patterns demonstrated that all diffraction peaks of the prepared sample are well-indexed with the standard spectrum diffraction peaks corresponding to the (100), (002), (101), (102), (110), (103), (200), (112), (201), (004), and (202) planes of ZnO (JCPDS no. 36–1451). The characteristic peaks located at 27.4° can be well-ascribed to the (002) planes of pure g-CN. Likewise, pronounced peaks at 27.4° were observed for P-CN [56]. The evident attenuation of the (002) peak intensity of P-CN reflected the heteroatom (P) substituted on the tri-s-triazine unit structure of CN nanosheets [53]. Hence, the intensity reduced while P-atoms were incorporated. It was mainly initiated by reducing the volume of piled carbon nitride interlayers and minor planar size of the constant tri-s-triazine unit disordered by P atoms [57]. The result obtained diffraction peaks corresponding to the (002), (100), (002), (101), (102), (110), (103), (200), (112), (201), (004), and (202) planes of the ZnO HPs/P-CN composite. It confirmed the formation of the ZnO HPs/P-CN composite.

Figure 1B represents the FTIR spectra, which characterized the functional groups and surface information of ZnO, g-CN, P-CN, and the ZnO HPs/P-CN composite. The foremost vibrational frequency (M-O) in ZnO was at 886 cm^−1^ owing to the Zn–O lattice [58]. P-CN kept similar vibration modes to g-CN, thus signifying that the P-doped 3D wrinkles structure array did not terminate the essential chemical structure of g-CN. The characteristic g-CN heterocycles in P-CN had stretching modes between 900 and 1700 cm^−1^ [59]. The peak M-O in the ZnO HPs/P-CN composite at 886 cm^−1^ was vibrational frequency owing to the Zn–O lattice and P-CN displayed peaks around between 900 and 1700 cm^−1^ in the ZnO HPs/P-CN composite.

**Figure 2:**
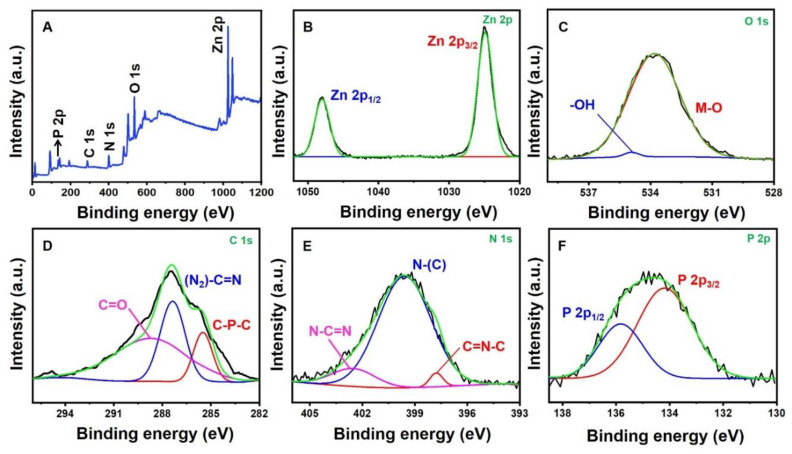
(**A**) A wide XPS survey spectra and fitting curves of high-resolution (**B**) Zn 2p, (**C**) O 1s, (**D**) C 1s, (**E**) N 1s, (**F**) P 2p of ZnO HPs/P-CN composite.

The XPS survey spectrum of the ZnO HPs/P-CN composite is shown in Figure 2A, where the peaks of Zn 2p, O 1s, C 1s, N 1s, and P 2p can be observed. Figure 2B represents a broad extended peak for Zn 2p_3/2_ and Zn 2p_1/2_ at a spectrum range of 1024.8 eV and 1047.9 eV, illustrating the presence of Zn lattice deities. Figure 2C shows that the O 1s spectrum attained at 533.9 eV could be ascribed to lattice oxygen as peaks of Zn–O bond. However, the weaker peak at 534.9 eV was related to oxygen in –OH species on the surface of ZnO. In the C 1s XPS spectrum (Figure 2D), the strong peak at a binding energy of 287.4 eV can be ascribed to carbon and the formation of defect-containing sp^2^-hybridized carbon atoms. The C 1s spectrum can be fitted with three peaks: C=O (288.8 eV), (N_2_)–C=N (287.3 eV), and C–P–C (298.5 eV). Where the N 1s spectrum peak (Figure 2E) is a mixture of three adjacent peaks in which the strong peak attained at 399.6 eV is due to the Sp^2^ hybridization of the triazine rings of N–(C) and the other two peaks are due to tertiary nitrogen radical deities and surface amino groups such as N–C=N and C=N–C compositions [60]. Where the doping of heteroatom sourced phosphorous P 2p signal (Figure 2F) is ascribed at a peak potential of 134.7 eV, which is an enclosure of two adjutant peaks attributed to the variation in P–N species of P 2p_3/2_ and P 2p_1/2_, this variation in chemical composition indicates that the C atoms were replaced by P forming a P–N bonding, illustrating the P-CN structure [60,61,62].

**Figure 3:**
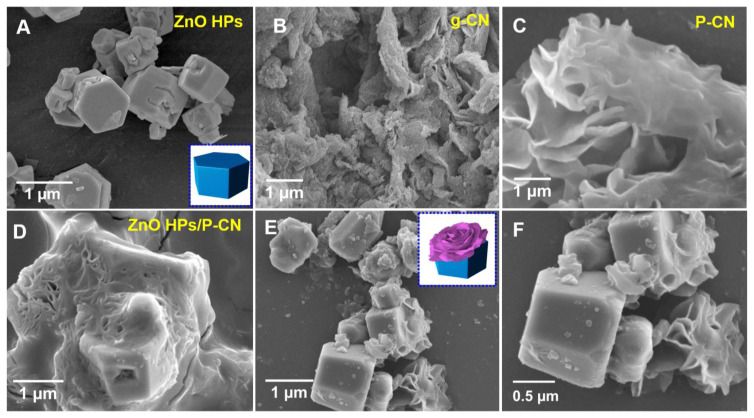
SEM images of (**A**) ZnO HPs (inset: hexagonal prism), (**B**) g-CN, (**C**) P-CN, and (**D**–**F**) ZnO HPs/P-CN composite (inset: wrinkle-like P-CN decorated on hexagonal prism).

**Figure 4:**
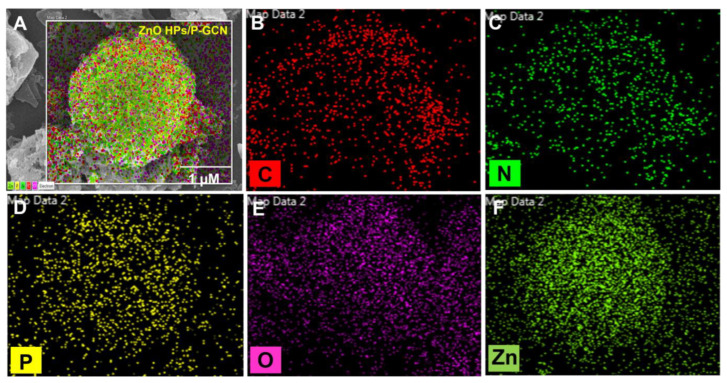
(**A**–**F**) Elemental mapping of ZnO HPs/P-CN composite.

SEM was adopted in observing the microscopic morphologies of the ZnO HPs/P-CN composite, as presented in Figure 3. As shown in Figure 3A, the ZnO material displays a hexagonal prism-like morphology with a smooth surface, with the inset image showing the hexagonal prism model. The pure g-CN (Figure 3B) was made of a nanosheets-like structure with wrinkles and stacking at the surface. It was observed that the P-CN (Figure 3C) possessed a 3D wrinkle-like morphology. The SEM of Figure 3D–F reveals the P-CN was well-dispersed on the ZnO HPs without agglomeration. This morphology would accelerate the transfer of electron rate and improve electrochemical properties. The elemental mapping images (Figure 4A–F) further confirmed the presence and homogeneous distributions of Zn, O, P, C, and N elements in the ZnO HPs/P-CN composite. The above physical characterization results were evident for the justification of ZnO HPs/P-CN composite formation.

### 3.2. Electrochemical Performance of ZnO HPs/P-CN Composite

#### 3.2.1. Electrochemical Characterization

**Figure 5:**
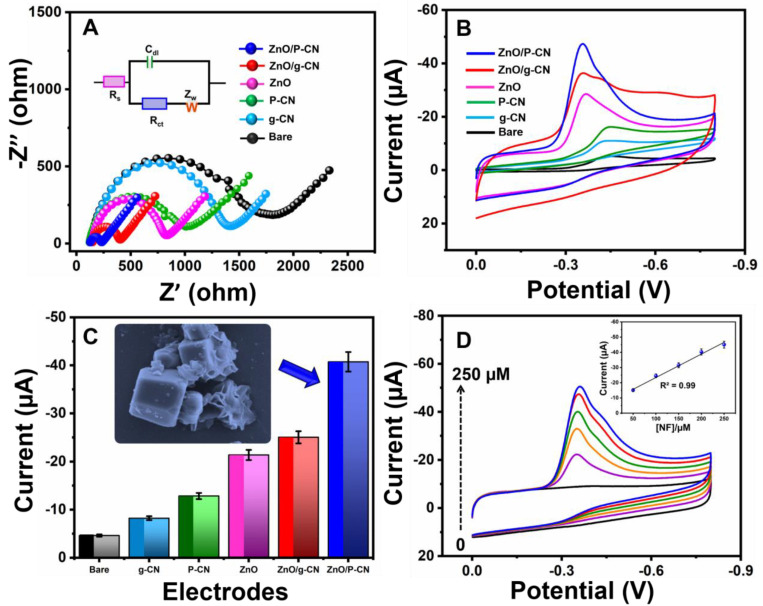
(**A**) EIS of bare GCE, g-CN/GCE, P-CN/GCE, ZnO HPs/GCE, ZnO HPs/g-CN/GCE, and ZnO HPs/P-CN/GCE (inset: Randles equivalent circuit). (**B**) CVs of unmodified and modified GCEs in 0.05 M PB presence of 200 µM NF. (**C**) Bar diagram for electrodes versus current from (**B**). (**D**) CVs of different concentrations of NF (50–250 µM) at ZnO HPs/P-CN/GCE in 0.05 M PB (inset: calibration plot of [NF]/μM vs. current (μA)).

Electrochemical impedance spectroscopy (EIS) was employed to expose essential resistivity and concourse changes among modified and unmodified electrodes. Figure 5A demonstrates EIS curves derived for bare GCE, g-CN/GCE, P-CN/GCE, ZnO HPs/GCE, ZnO HPs/g-CN/GCE, and ZnO HPs/P-CN/GCE in 0.1 M KCl consisting of 5 mM Fe(CN)_6_^3−/4−^. The Randles equivalent circuit model was utilized for investigating the electron charge transfer (R_ct_), assembly of series resistance (R_s_), double layer capacitance (C_dl_), and Warburg diffusion (Z_w_) which was attained to suit the experimental data and perform analysis, as shown in Figure 5A. As per the results, the bare GCE (1682.9 Ω) showed a larger R_ct_ value associated with the modified electrodes. Now, the R_ct_ value of the ZnO HPs/P-CN/GCE (102.2 Ω)-modified electrode seemed to be a smaller semicircle than the g-CN/GCE (1261.6 Ω), P-CN/GCE (884.8 Ω), ZnO HPs/GCE (677.8 Ω), and ZnO HPs/g-CN/GCE (253.4 Ω), which shows that owing to a smaller contact, less charge transfer impedance has happened in the modified electrodes. From the analysis results, the fabricated ZnO HPs/P-CN/GCE has an efficient electrochemical activity towards the determination of NF [63].

#### 3.2.2. Electrochemical Behavior of Electrodes

**Scheme 2:**
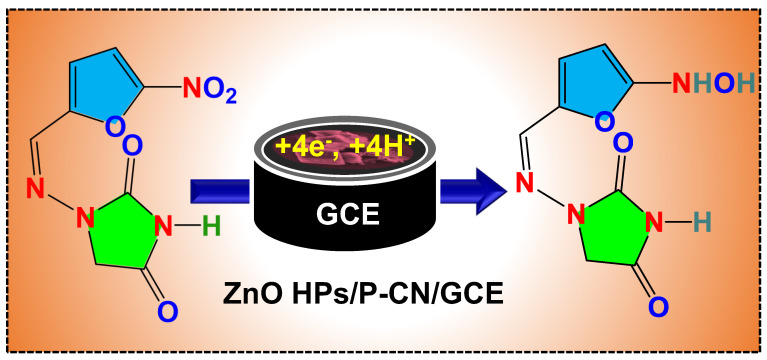
The electrochemical reduction mechanism of NF.

The electrochemical behavior of the modified electrode with and without the addition of NF in 0.05 M PB (pH 7.0) was investigated by CV at the scan rate of 50 mV/s. This can be seen in Figure 5B, where a reduction peak of NF was observed at bare GCE, ZnO HPs/GCE, g-CN/GCE, P-CN/GCE, and ZnO HPs/P-CN/GCE, which is due to the direct electrocatalytic reduction of NF to phenyl hydroxylamine with the 4e−/4H+ transfer process. During this reverse scan, there was not any anodic peak observed related to the reduction peak of NF, indicating that the catalytic reduction of NF is an irreversible process [64]. Among these electrodes, the ZnO HPs/P-CN/GCE showed a sharp and well-distinct reduction response of −45.07 µA at about −0.35 V. The reduction response of NF at the ZnO HPs/P-CN/GCE is relatively 8.82-, 4.95-, 3.17-, 1.9-, and 1.62-folds greater than those of bare GCE, g-CN/GCE, P-CN/GCE, and ZnO HPs/GCE, respectively (Figure 5C). The preference of the electrochemical performance of the ZnO HPs/P-CN/GCE compared to the other electrodes was credited to numerous advantages. Especially, the enhanced activity may be owed to the synergistic effect between ZnO HPs and P-CN in the composite towards NF reduction. Likewise, the ZnO HPs/P-CN composite provides a more active site, a large specific surface area, improved electrical conductivity, and favorable transportation. The proposed electrochemical mechanism of the ZnO HPs/P-CN modified electrode to NF was stated based on the literature (Scheme 2) [21,22,23]. The results proved that the best and good electrocatalytic properties were observed with the ZnO HPs/P-CN modified electrode.

#### 3.2.3. Effects of Concentration, pH, and Scan Rate

The electrochemical performance of ZnO HPs/P-CN/GCE was evaluated by the CV approach with various concentrations of NF in 0.05M PB (pH 7.0) at a scan rate of 50 mV/s (Figure 5D). The electrochemical reduction current increased linearly with increasing target analyte concentrations ranging from 50 to 250 µM. The linear regression equation was expressed as Ipc (µA) = −0.1511 [NF] (µM) − 8.648 with a correlation coefficient (R^2^) of 0.99 (Figure 5D inset image). These results suggest that the proposed ZnO HPs/P-CN/GC shows an outstanding catalytic performance towards detecting NF.

**Figure 6:**
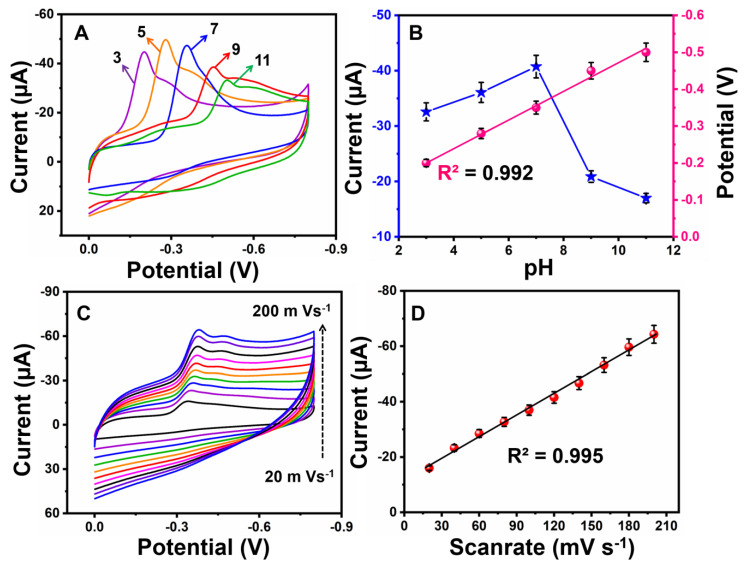
(**A**) CVs of ZnO HPs/P-CN/GCE in 0.05M PB with different pH containing 200 μM NF. (**B**) Effect of pH value on the I_pc_ and E_pc_. (**C**) CVs of different scan rates (20–200 mV s^−1^) of ZnO HPs/P-CN/GCE. (**D**) Corresponding linear plot of scan rates versus current.

The effect of the pH on the reduction response of NF was examined from pH 3.0 to 11.0 in 0.05 M PB using the ZnO HPs/P-CN/GCE (Figure 6A). As seen, the reduction current increased with increasing pH starting with 3.0 towards 7.0 and, beyond that, decreased when the pH was more than 7.0 for NF reduction, which is in agreement with the literature. We noted that the peak potential shifted for NF reduction towards more cathodic values with pH values increasing, which is due to the direct association of protons in the electrode reaction. In addition, a higher reduction current was observed at pH 7.0 (Figure 6B). Moreover, a linear relationship was observed between the reduction peak potential (E_pc_) and pH values, as the following equation: Epc (µA) = y = −0.0385 pH − 0.0865 (R^2^ = 0.992) (Figure 6B). A slope of 38.5 mV for each pH value was obtained, which is closer to the theoretical Nernstian value of 59 mV pH-1, implying an electron transfer number convoyed by an equal quality of electrons and protons on the surface of ZnO HPs/P-CN/GCE. Hence, the 0.05M PB at pH 7.0 was chosen as the effective channel for subsequent NF electrochemical detection.

The influence of the scan rates on the electrochemical reduction response of NF at the ZnO HPs/P-CN-modified GCE was derived by the CV approach. Figure 6C displays the CV signals of ZnO HPs/P-CN/GCE in a 0.05 M PB (pH 7.0) containing 100 μM NF at different scan rates. The cathodic peaks current was amplified with the increasing scan rate in a range of 20–200 mV/s. A linear relationship between the reduction peak current and scan rate with a regression equation of I_pc_ (μA) = −0.2605 υ (mV/s) −11.633 (R^2^ = 0.995) was observed (Figure 6D). The linear dependencies of the reduction peak currents on the sweep rate indicate that the ZnO HPs/P-CN-modified electrode is a process controlled by adsorption [65].

#### 3.2.4. Electrochemical Detection of the Sensor

**Figure 7:**
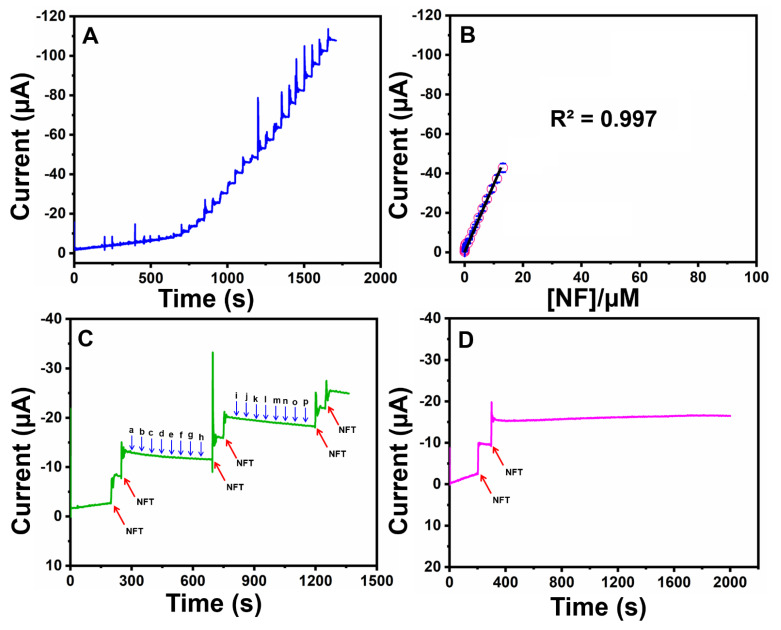
(**A**) The i-t responses of NF at the ZnO HPs/P-CN/GCE for the various additions of NF from 0.01 to 914 μM into constantly stirred 0.05 M PB (pH = 7.0). (**B**) Calibration plot of the concentration (NF/µM) vs. current (µA). (**C**) Selectivity and (**D**) stability of the ZnO HPs/P-CN/GCE.

The quantitative analysis of NF on ZnO HPs/P-CN-modified GCE was carried out through the amperometry (i-t) method based on the optimal parameters. The reduction peak currents were found to be enhanced linearly with injection in the concentration of NF from 0.01 to 914 µM, as illustrated in Figure 7A. Our proposed electrochemical sensor detected NF with a linear region of concentration in 0.01–111 µM with a R^2^ value of 0.997 (Figure 7B). The LOD and sensitivity were determined as 2 nM and 4.62 µA µM^−1^·cm^2^ for the ZnO HPs/P-CN-modified electrode towards NF detection, respectively. The electrochemical performance of ZnO HPs/P-CN/GCE was compared with other previous NF sensors as shown in Table 1. These results demonstrate that the ZnO HPs/P-CN sensor exhibits greater electrochemical reduction ability towards NF sensing.

#### 3.2.5. Specificity and Stability of the Sensor

The ZnO HPs/P-CN-modified electrode behavior toward NF sensing was inspected using the i-t technique in the occurrence of various potentially interfering species (Figure 7C). The anti-interference ability of the ZnO HPs/P-CN-modified electrode was examined towards NF with possible interferents, including furazolidone (a), nitrofurazone (b), 4-nitrophenol (c), chloramphenicol (d), flutamide (e), hydroquinone (f), catechol (g), uric acid (h), hydrogen peroxide (i), hypochlorous acid (j), sodium (k), nitrite (l), iron (m), mercury (n), copper (o), and lead (p). A 100-fold excess of a-g and 200-fold of h-p was added individually in the 0.05 M PB (pH 7.0) with 100 μM NF. There were almost no significant changes in NF response for potentially interfering species. The obtained results indicate that the ZnO HPs/P-CN-modified electrode has an outstanding selectivity towards NF sensing. The operational stability of the ZnO HPs/P-CN-modified electrode was evaluated with an injection of 100 µM NF (Figure 7D). The reduction current for NF maintains (±) 2.6% after 2000 s of the fabricated ZnO HPs/P-CN modified electrode. The above outcome reveals that the ZnO HPs/P-CN-modified electrode possesses superior selectivity and excellent stability.

#### 3.2.6. Detection in Real Samples

**Figure 8:**
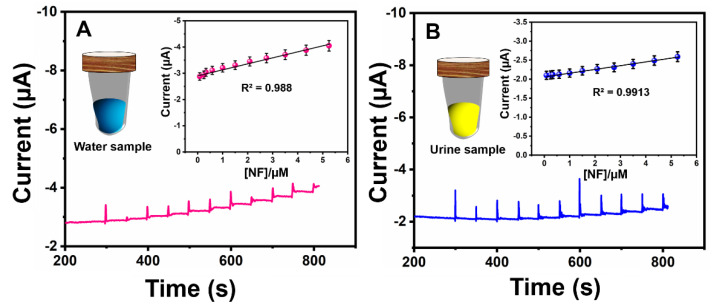
The amperometric real-world sample analysis responses in (**A**) water and (**B**) human urine samples of ZnO HPs/P-CN/GCE for the various additions of NF into constantly stirred 0.05 M PB (pH = 7.0).

The feasibility of the fabricated ZnO HPs/P-CN sensor was confirmed by its real-time analysis of NF in environmental and biological samples. The river water sample was used as the environmental and the urine samples were used as biological samples for detection of NF via the i-t technique. However, NF was not detected in the environmental and biological samples. To assess the practicability of the ZnO HPs/P-CN sensor, a standard solution with a known concentration of NF was spiked into the environmental and biological samples. The recovery rate of NF was estimated by a typical addition procedure. As displayed in Figure 8A,B, reduction peak current intensity increased step-wise with the addition of NF-spiked water, and the biological samples were in the range from 0.083 to 6 µM with a rapid response of 3 s. Based on the above, the fabricated ZnO HPs/P-CN sensor can accurately, and satisfactorily detect the NF in water and biological samples, which has confident application prospects in real-time applications.

## 4. Conclusions

In brief, we have effectively synthesized a ZnO HPs/P-CN composite for selective electrochemical detection of NF. The formation of the ZnO HPs/P-CN composite was confirmed by including XRD, FTIR, SEM, and elemental mapping. The ZnO HPs/P-CN composite modified electrode showed lower R_ct_ values than other modified electrodes with significant performance to the reduction of NF compared to the other electrodes. Particularly, the ZnO HPs/P-CN composite modified electrode displayed an advantage in its outstanding low detection limit, wide linear range, good sensitivity, high stability, and good selectivity. More importantly, the ZnO HPs/P-CN-based sensor was successful in detecting NF in environmental and human samples with high accuracy.

## Figures and Tables

**Table 1:** Analytical performance of the ZnO HPs/P-CN-modified electrode for NF determination compared with the previous literature.

Electrode Materials	Quantitative Method	Linear Range (µM)	Sensitivity (μA μM^−1^ cm^−2^)	LOD (µM)	Ref.
ErGO-CNT	i-t	0.005–2.81	36.3	0.0187	[9]
LuV/GR	i-t	0.008–256	1.709	0.001	[10]
NiSnO	i-t	0.0066–466.6	–	0.003	[12]
rGO/Fe_3_O_4_ NRs	DPV	0.1–100	–	0.083	[14]
NiO/BCN	i-t	0.05–230	1.15	0.01	[15]
BDDFE	SWV	0.497–5.66	–	0.0082	[21]
NiFe/*f*-MWCNT	DPV	0.1–352.4	11.45	0.03	[22]
MgFe_2_O_4_	DPV	3–302	–	0.033	[23]
**ZnO HPs/P-CN**	**i-t**	**0.01–111**	**4.62**	**0.002**	**This Work**

## Data Availability

Available on request.
